# Tribological Properties of Ni-P/Si_3_N_4_ Nanocomposite Layers Deposited by Chemical Reduction Method on Aluminum Alloy AW-7075

**DOI:** 10.3390/ma13245797

**Published:** 2020-12-18

**Authors:** Kazimierz Czapczyk

**Affiliations:** Faculty of Ocean Engineering and Ship Technology, Gdansk University of Technology, 80-233 Gdansk, Poland; kazimierz.czapczyk@pg.edu.pl; Tel.: +48-58-3472713

**Keywords:** aluminum alloy, nickel, nanocomposite layers, tribology

## Abstract

The article presents the results of tribological tests of Ni-P/Si_3_N_4_ nanocomposite and Ni-P nickel layers deposited on the AW-7075 aluminum alloy by chemical reduction method, and the AW-7075 alloy without coating. Nanocomposite layers were produced using Si_3_N_4_ siliconnitride in the form of a polydisperse powder whose particle sizes ranged from 20 to 25 nm. The influence of the content of the dispersion phase layer material on the abrasive wear, which was determined as the “ball on disc” method, was analyzed. Surface topography was examined by the contact method using a profilometer. The purpose of introducing Si_3_N_4_ particles into the Ni-P layer was to increase the wear resistance of AW-7075 aluminum alloy parts with an embedded nanocomposite coating. Based on the obtained test results, it was found that the Ni-P/Si_3_N_4_ layers are more resistant to wear than the Ni-P layers and the AW-7075 alloy layers, and are a good barrier against abrasive wear at various loads and environmental conditions.

## 1. Introduction

AW-7075 aluminum alloy is currently an important construction material in various industries due to its lower weight and better corrosion resistance compared to steel. In addition, some aluminum alloys have similar strength properties to some structural steels—e.g., C45 used on gear wheels. For this reason, the AW-7075 alloy is often found in shipbuilding, aviation, and the automotive industry and also finds applications, e.g., in gears, hull structures, etc. Unfortunately, aluminum alloy components are characterized by less resistance to abrasion due to friction than steel parts. One of the possibilities to improve tribological properties is surface treatment consisting of depositing the appropriate layer. Aluminum alloys can be coated with various coatings for technical applications. The substrate with a deposited coating makes a certain areological system that requires the use of an appropriate connection between the elements, allowing for structural adjustment [1,2]. The right choice of a coating material in terms of hardness is very important because it affects its resistance to abrasive wear [2,3]. Currently, nanocomposite coatings are of great interest, which, when used on aluminum alloys, could significantly increase the wear resistance of the surface layer of a given part [4]. Most often, such coatings consist of ceramic or polymer particles or nanoparticles in a metallic matrix of nickel–phosphorus alloy. Particles that are dispersion phases can be, e.g., SiC, Si_3_N_4_, Al_2_0_3_, PTFE, diamond, or other [5]. Coatings with ceramic particles have high hardness and wear resistance; therefore, they affect the mechanical and tribological properties of coatings. However, a coating with nanoparticle content will have different properties when compared to the same coating material with microparticle content, because the size contributes, for example, to surface roughness changes. Further improvements of properties, including resistance to abrasive wear, are possible through the use of heat treatment. In addition, overly high content of nanoparticles contributes, among others, to reducing hardness and tribological properties. Nanoparticles play a very important role in improving the properties of coatings, but only the proper selection of the dispersion phase, particle size, their content, and possible heat treatment will allow obtaining the optimal coating for given operating conditions [6,7,8,9,10,11,12,13,14,15,16,17,18,19,20,21,22,23,24,25,26,27,28,29,30,31,32,33,34,35,36].

The main aims of testing electroless coatings such as Ni–P are to increase mechanical properties and define how much the layers may increase the wear resistance of different machine elements. For example: preliminary results of tribological tests in the three balls and cone method showed that the Ni-P/Si_3_N_4_ layers deposited in the bath with the addition of 10 g/dm^3^ of Si_3_N_4_ nanoparticles on the 7075 aluminum alloy are characterized by better tribological properties compared to the Ni-P layers. Ni-P/Si_3_N_4_ nanoparticles contribute primarily to increasing the hardness of the surface, and also present good abrasion resistance [37]. In other similar studies, where the substrate was iron, a significant increase in microhardness and very good results in terms of abrasion in pin-on-disc tests were also obtained [4]. On this basis, it was found that nanocomposite layers are a good material for tribological research when used on aluminum alloy AW-7075, which is currently being attempted to be used more widely for moving machine parts. Additionally, surface topography and morphology analyses were performed to confirm the effect of Si_3_N_4_ nanoparticles on the structure of the coating material and the friction coefficient parameter in tribological studies.

## 2. Materials and Methods

The subjects of the study were aluminum alloy and coatings of different chemical compositions and different thicknesses. The samples were made of AW-7075 aluminum alloy, and they were prepared as discs with a thickness of 7 mm and a diameter of 50 mm. On their substrates were deposited the Ni-P nickel and Ni-P/Si_3_N_4_ composite layers by chemical reduction method. The chemical composition of the alloy is specified in Table 1.

Samples made of AW-7075 alloy were subjected to preparatory processes before coating deposition. First, the samples were degreased in an organic solvent, digested in an alkaline solution, and also galvanized in a multi-component solution. Then, for the deposition of layers by means of the chemical reduction method, a bath was prepared with the following composition: NiSO_4_, NaH_2_PO_2_ reducer, and C_2_H_4_OHCOOH buffer, which stabilized the pH (4.3–4.6). The bath temperature during the deposition process was approximately 363 K. Nanocomposite layers were obtained by introducing the polydisperse Si_3_N_4_ powder as a dispersion phase with a particle size of 20–25 nm. The content of Si_3_N_4_ nanoparticles in the baths was 2 g/dm^3^ or 5 g/dm^3^. Deposition of composite coatings was carried out by simultaneous deposition on Ni-P layers and Si_3_N_4_ nanoparticles. Ultrasonic mixing before the deposition process and mechanical mixing during layer deposition were also used to ensure adequate particle dispersion, prevent sedimentation, and obtain a homogeneous suspension and efficient transfer of the reinforcing phase [38]. All coating thicknesses were different and were 10 ± 2, 20 ± 2, and 30 ± 2 µm based on the adjusted deposition times of 60, 120, and 180 min.

The surface morphology and topography analysis was performed using a Tescan Vega 5135 scanning electron microscope with an EDS PGT Prism 200 X-ray spectrometer (which allowed checking the contents of nickel, phosphorus, and silicon, without nitrogen) and AltiMap Premium 7.1.7037 profilometer. Surface topography tests were carried out individually and separately on each sample. Topography sites were selected in the central parts of the samples, and the tested surface was a 5 × 5 mm square. The images were used to determine, among others, areas of hollows, elevations, bulges, and the detection of possible damage or abnormalities of the coating material before tribological tests, because its type and thickness affect the surface structure. In contrast, the morphology of each sample was examined multiple times in several places, and microscopic images are shown in Figures 2 and 3.

Microhardness tests of alloy and layer material were carried out on the PICODENTOR HM500 device. Measurements were made using the Vickers method with indenter loading of 300 mN in the period of 20 s, while maintaining this force for 5 s. The microhardness test was carried out without piercing the material of the tested layers. Averaged measurement results are presented in Table 7. Five measurements were taken from each sample. The tribological properties of the layers deposited on the AW-7075 substrate were tested by the ball-on-disc method using the laboratory tribotester of the Institute of Precision Mechanics in Warsaw and tribotester of Poznan University of Technology.

Tribological tests were carried out in accordance with the current standards PN-EN 1071-13 and PN-EN 1071-12. The measurements were divided into two parts and were carried out at different unit pressures and under different conditions. The wear marks were subjected to detailed microscopic analyses, which were carried out using the Keyence VHX 5000 optical microscope, and the widths of the resulting cracks were adopted as the wear criterion—in accordance with PN-EN 1071-13. In addition, in the second part of the tests, acoustic emission signals and friction force were recorded, which, combined with microscopic images, were used to determine the wear resistance of the layers.

Apart from that, for all steps there was also calculated the rough estimation of contact pressure (using Hertzian contact theory) for the case of pressing the ball against a flat surface (Table 2 and Table 3).

In the first part, in the ball-on-disc study at the Institute of Precision Mechanics in Warsaw they was divided it into two stages that are described in Table 2.

In the first part, in both stages, the specimen surfaces were spray-coated with a thin layer of MoS_2_ dry lubricant to prevent damage to the specimen surfaces, and the head with the counterspecimen was mounted. For comparative purposes, a tribological test was also carried out on a sample from the AW-7075 alloy in technically dry conditions.

The initial stage was aimed at determining the possibility of conducting this type of testing, and its continuation, by carefully observing the behavior of specimens from aluminum alloy without coatings and with coatings during measurements. After testing, the specimens were subjected to microscopic examination to accurately determine the widths of the cracks and surface wear. In addition, the results obtained in the first part of the study also allowed the second part to be carried out, which was carried out in a different environment and with different parameters on a laboratory machine at Poznan University of Technology.

In the second part, the ball-on-disc study at the Faculty of Mechanical Engineering and Management of Poznan University of Technology was carried out in two stages on a BRUKER UMT TriboLab laboratory machine, which additionally allowed recording of, among others, the values of friction forces and acoustic emission. The tests were carried out with the use of GL-4 75W/90 semi-synthetic gear lubricant. The samples were placed in a special oil-filled tub; then a counterspecimen was assembled to which the pressure appropriate to the tested surface was applied.

**Figure 1:**
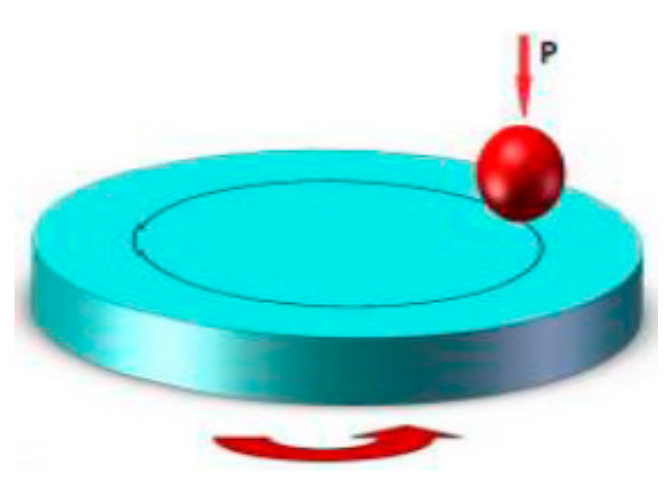
Scheme of the ball-on-disc test [7].

The parameters (in the second part) are presented in Table 3. The general scheme of tribological test for the first and second part is presented in Figure 1.

After the measurements were carried out, the specimens were again subjected to microscopic examination in order to accurately check the condition of the surface and the widths of the resulting cracks. In addition, the parameters registered by the machine (friction force, normal force, acoustic emission, and automatically calculated friction coefficient value) were analyzed.

## 3. Results

The AW-7075 aluminum alloy substrate itself, on which Ni-P and Ni-P/Si_3_N_4_ nanocomposite layers were deposited, had the same mechanical properties and chemical composition.

### 3.1. Characteristics of Layers

**Figure 2:**
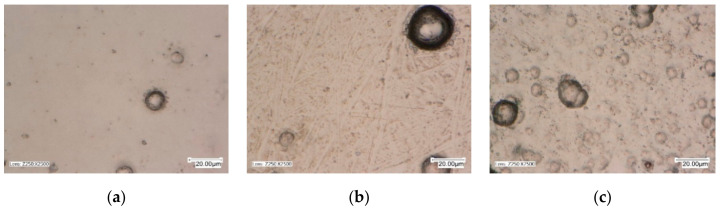
Morphology of Ni-P and Ni-P/Si_3_N_4_ layers; (**a**)—Ni-P, (**b**)—Ni-P/Si_3_N_4_ (2 g/dm^3^), (**c**)—Ni-P/Si_3_N_4_ (5 g/dm^3^).

The results of morphology tests for Ni-P layers and layers with the Si_3_N_4_ dispersion phase are presented in Figure 2. The matrix of composite material of layers produced by chemical reduction was a solid solution of phosphorus in Ni-P nickel containing 6% by mass P. According to [3,11,14], the structure of the coating depends on the type of layer and its composition. In the case of a composite material, the structure is determined by the structure of the matrix material, the type of dispersion ceramic phase, its structure, the degree of fragmentation, and the content in the material. In addition, the content of the dispersion ceramic phase in the composite coating depends on the concentration of the powder in the galvanic bath. When the Si_3_N_4_ content in the bath increases, the number of particles that are incorporated into the material of the deposited coating increases. The increase in the content of Si_3_N_4_ nanoparticles contributes to the formation of highly concentrated sites.

**Figure 3:**
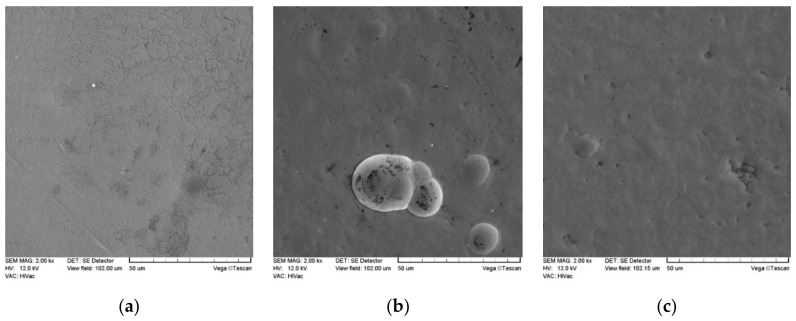
SEM images of the morphology of Ni-P and Ni-P/Si_3_N_4_ layers; (**a**)—Ni-P, (**b**)—Ni-P/Si_3_N_4_ (2 g/dm^3^), (**c**)—Ni-P/Si_3_N_4_ (5 g/dm^3^).

**Figure 4:**
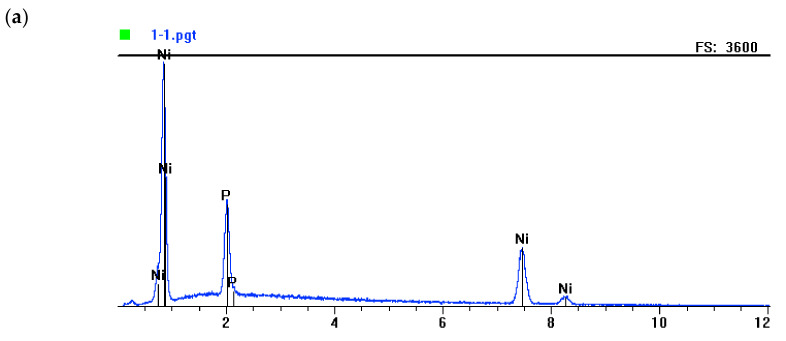

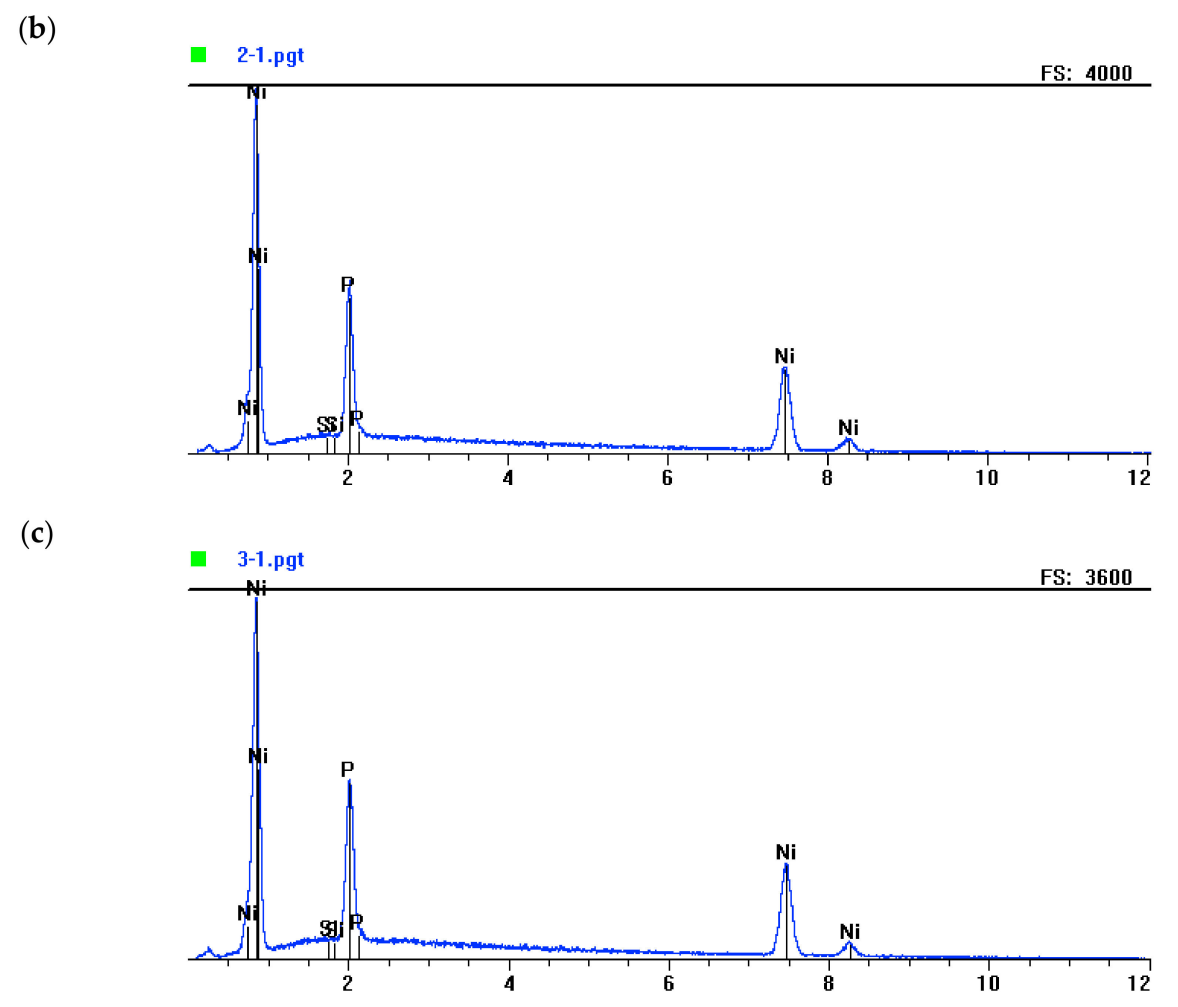
EDS analysis of chemical composition of Ni-P and Ni-P/Si_3_N_4_ layers; (**a**)—Ni-P, (**b**)—Ni-P/Si_3_N_4_ (2 g/dm^3^), (**c**)—Ni-P/Si_3_N_4_ (5 g/dm^3^).

The morphology test results for Ni-P layers and phase layer-dispersive Si_3_N_4_ are shown in Figure 2 and Figure 3. The results of EDS tests are shown in Figure 4. and the EDS graphs directly correspond to specific values in Table 4—the part called “chemical elements.” Apart from that, the same values (in Table 4) correspond also to the prepared galvanic baths where the layers were created.

The results of morphological and SEM tests confirm that all tested coatings, which are determined by the structure of the matrix material, are characterized by a homogeneous and compact structure. The structures of nanocomposite layers differ from the Ni-P layer due to the content of the dispersion phase. Compared to Ni-P layers without a built-in dispersion phase, the surfaces of Ni-P/Si_3_N_4_ layers are more corrugated and without gloss (qualitative results), but the differences are better noticeable in roughness measuring (quantitative results). Si_3_N_4_ nanoparticles are very small and are difficult to capture with a microscope. On the surfaces of composite layers, the agglomerates of Si_3_N_4_ nanoparticles are well anchored, and no material defects, such as discontinuities, losses, pores, and microcracks, have been observed.

**Figure 5:**
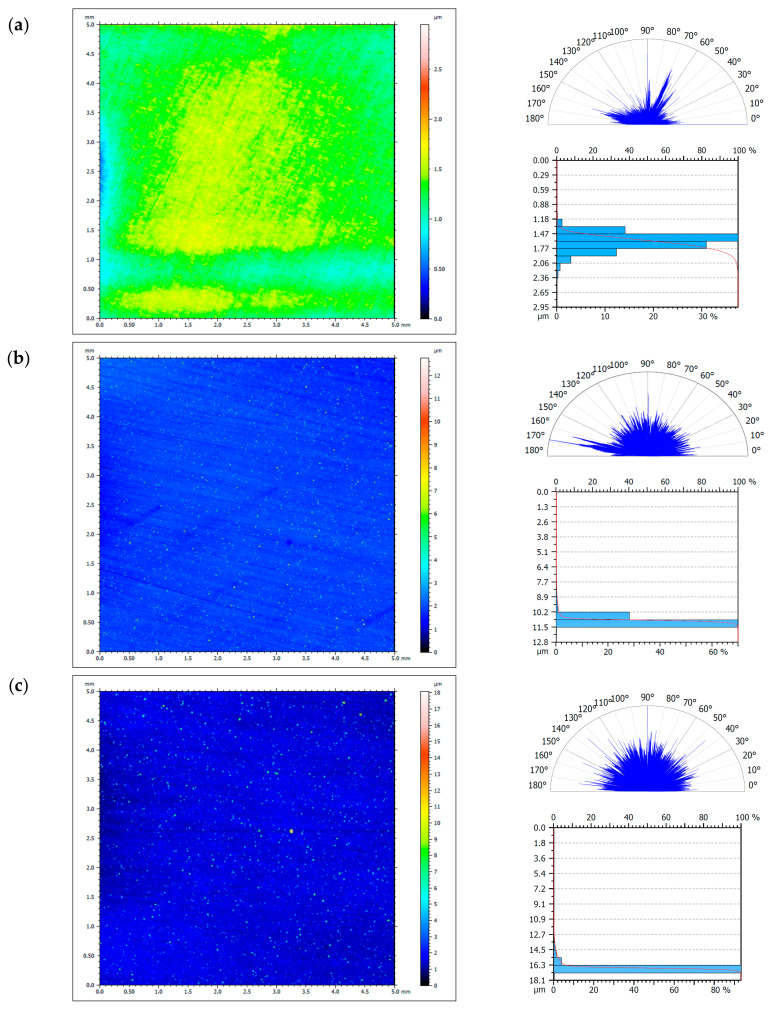

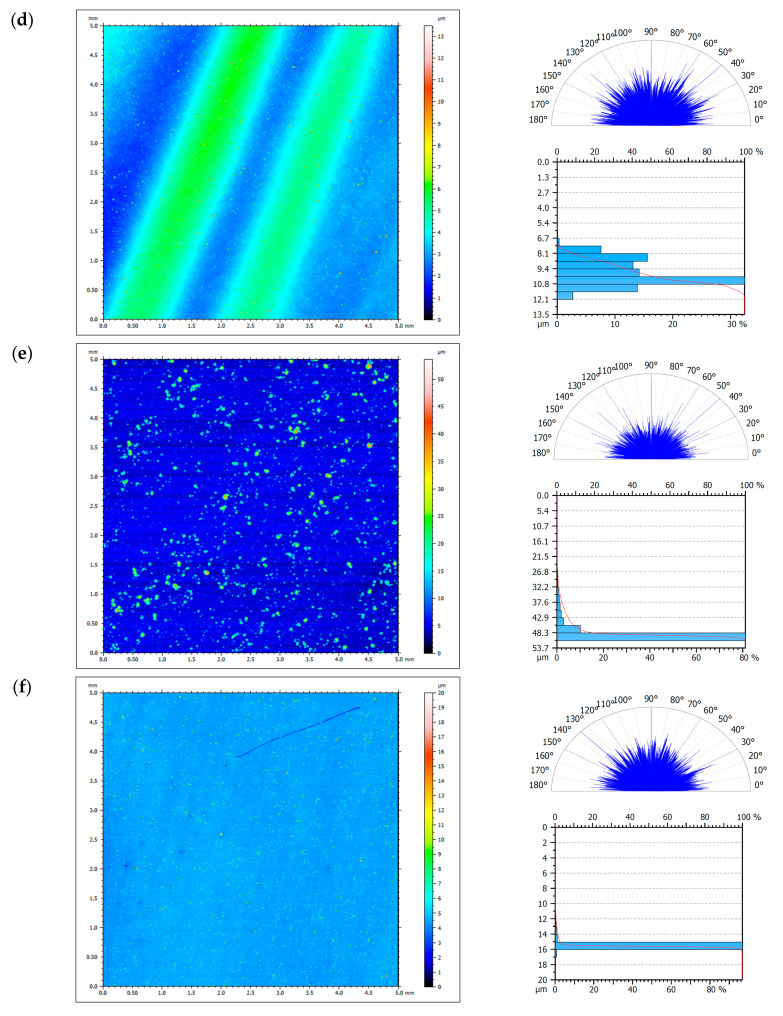
Surface topography of the AW-7075 alloy and Ni-P and Ni-P/Si_3_N_4_ coatings. ((**a**)—AW-7075, (**b**)—Ni-P(10 μm), (**c**)—Ni-P(20 μm), (**d**)—Ni-P(30 μm), (**e**)—Ni-P/Si_3_N_4_ (2 g/dm^3^), (**f**)—Ni-P/Si_3_N_4_ (5 g/dm^3^)).

Figure 5 shows two-dimensional images (the horizontal “x” and vertical “y” axes mean only vertical or horizontal sides of the tested field, presented in millimeter units: 0–5 (mm)) of the tested surfaces, diagrams of surface structure directions, and Abbott–Firestone curves (the axis “y” means “profile heights” and the “x” means “material percentage”) from profilometric tests for visual imaging and comparison that correspond to specific values of results of the roughness in Table 5, and the surface structures of the specimens in terms of their isotropy, along with various directions in Table 6.

For specimens with deposited coatings in two-dimensional images (Figure 5), brighter points were visible, which occurred in the form of “needles” and contributed to increasing the roughness (Table 5). All specimens with deposited Ni-P and Ni-P/Si_3_N_4_ layers were characterized by an isotropic structure except for a sample with a 30 μm thick Ni-P coating. This result was influenced by the clearly wavy shape of the surface (Figure 5d) obtained after machining. In the case of a specimen without a deposited layer, the surface was characterized by a mixed structure, despite its being very thoroughly polished to the so-called “smooth mirror” finish. The isotropy difference (Table 6) between the sample from the AW-7075 alloy and the samples with deposited layers was from 40 to 55.2% (not including the sample with Ni-P coating 30 μm thick due to surface shape error). Deposition of Ni-P and Ni-P/Si_3_N_4_ layers by chemical reduction method on a mechanically and chemically prepared substrate contributes to an increase in the surface isotropics of the samples.

### 3.2. Microhardness of Layers

**Figure 6:**
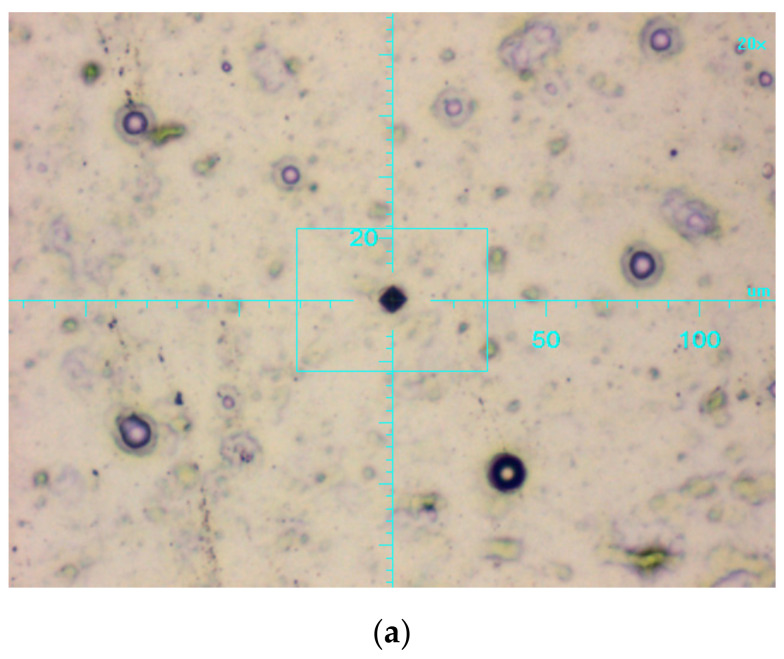

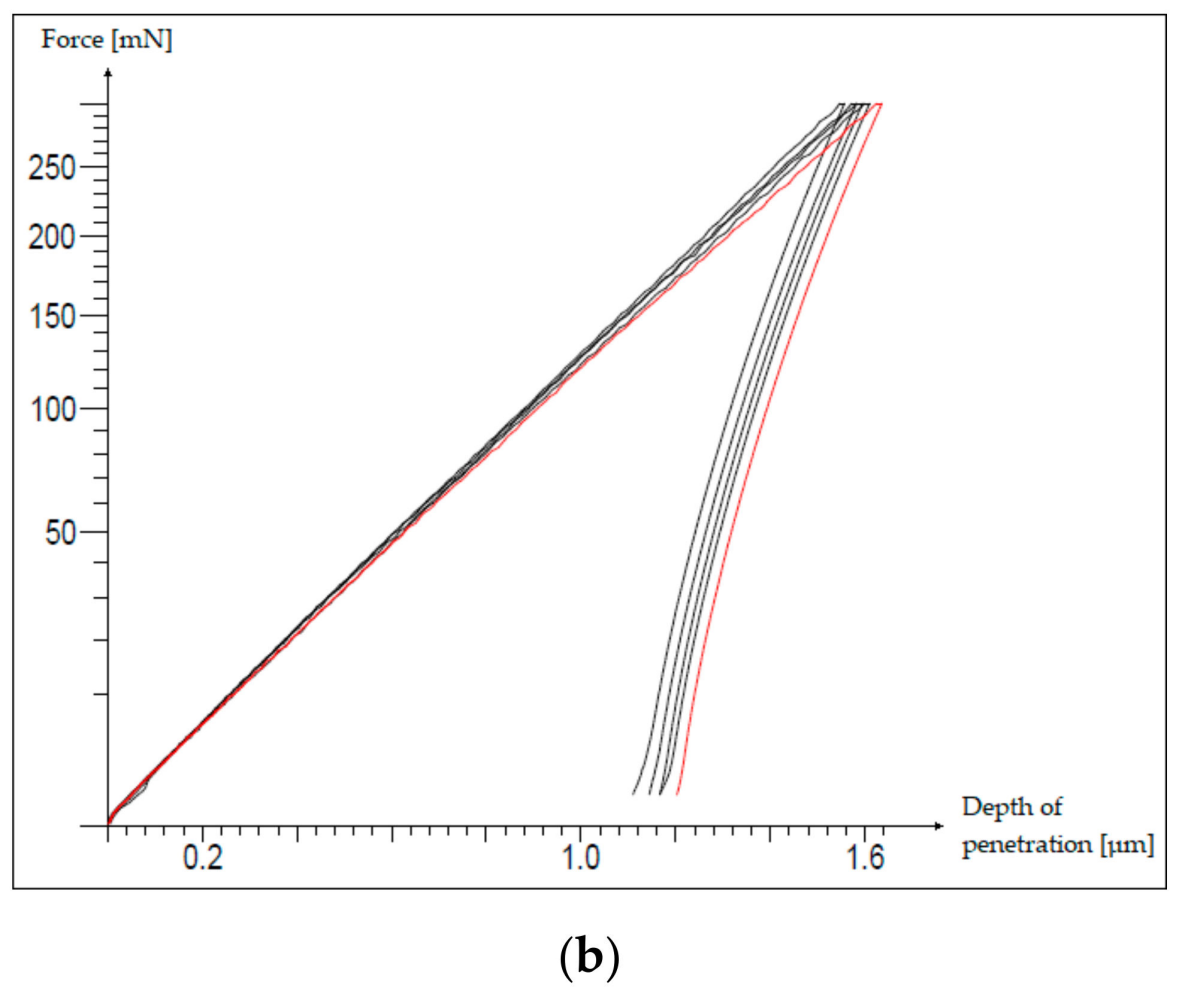
Print image at 20× magnification (**a**) and depth penetration diagram (**b**) of Ni-P/Si_3_N_4_ (2 g/dm^3^).

The second study tested the microhardness of the alloy and nickel and composite layers. The results are presented in Table 7 and Figure 6. The 10 µm Ni-P coating showed much higher hardness than the AW-7075 aluminum alloy itself. In contrast, coatings with a built-in Si_3_N_4_ dispersion phase showed the highest hardness for all previously tested AW-7075 samples and for a Ni-P coating without a dispersion phase, at the same thickness, i.e., 10 µm. However, the content of Si_3_N_4_ nanoparticles in the coatings was different, and it was noted that the content of the dispersion phase influences the increase in the hardness of the layer; however, the highest hardness was obtained for the content of 2 g/dm^3^, while for 5 g/dm^3^ the hardness began to decrease again. This relationship was noticed on all samples tested many times.

Figure 6 shows the image of print and penetration depth diagram obtained from the micro hardness tester Picodentor HM500. The results of the penetration depth for all layers were very similar to each other and amounted to approximately 1.2 μm, and for the AW-7075 alloy this value was higher and amounted to approximately 2.3 μm. For the coatings with the smallest thickness (10 μm), the penetration depths constituted only 12% of their thickness, and at the maximum load, this value temporarily increased to a maximum of 16%—that is visible on the diagram (Figure 6b). In this case, with very small deformations on the tested layers (Ni-P and Ni-P/Si_3_N_4_) with a thickness of 10 μm, the influence of the substrate was unnoticeable, which was also documented in another earlier study, examining the effect of the thickness of Ni-P layers (10, 20, 30 μm) on the penetration depth, which was still constant and repetitive [39].

### 3.3. Tribological Tests

Tests of surface resistance to abrasive wear by means of the ball-on-disc method using MoS_2_ dry grease:

Test results are presented in the order in which they were carried out. This section first presents the results of the preliminary tests—the first stage, and then of the second stage. The results of the preliminary tribological tests, which were carried out at a load of 7.5 N, a rotational speed of 2 s^−1^, and during 60 s (1 min), are presented in Figures 9 and 10. The wear diameters for all samples were 15 mm. The drawings show high resolution microscopic images in which crack widths or carved grooves with an accuracy of 0.1 µm were marked using specialized computer software.

**Figure 7:**
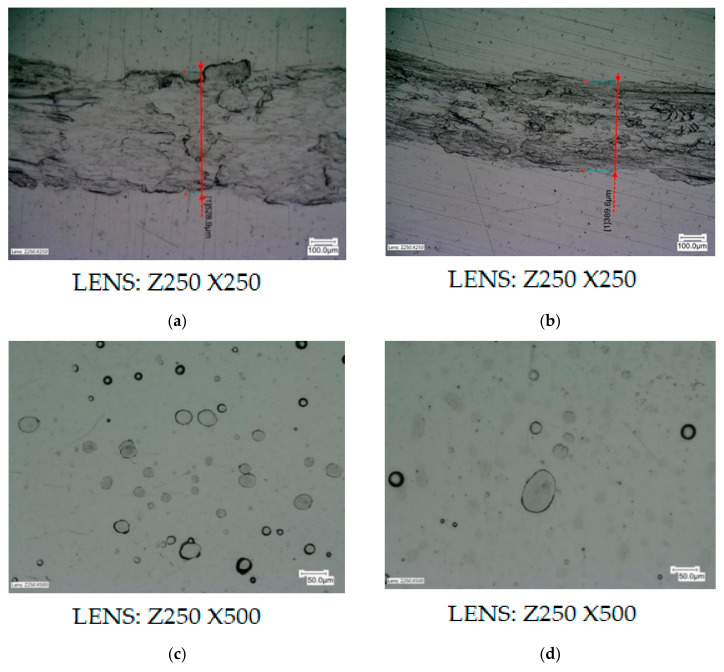
Samples of alloy AW-7075 without coatings and with Ni-P/Si_3_N_4_ layers. ((**a**)—AW-7075 without grease; (**b**)—AW-7075 with MoS_2_ grease; (**c**)—Ni-P/Si_3_N_4_ (2g) with MoS_2_ grease; (**d**)—Ni-P/Si_3_N_4_ (5g) with MoS_2_ grease; crack widths (µm): (**a**)—528.9, (**b**)—389.6, (**c**)—no scratches, (**d**)—no scratches).

Based on the results obtained from preliminary tribological tests, which are presented in Figure 7, it was noticed that in the case of samples from aluminum alloy AW-7075 without a deposited coating, after 1 min clear, wide, and uneven gouges in the material appeared in the form of grooves with a width of approximately 528.9 μm, characterized by surface damage in the form of losses and nicks. The use of a thin layer of MoS_2_ dry grease (ZEP Dry Moly), which was sprayed on the sample, reduced the grooves on the surface of the AW-7075 alloy, and the groove widths were approximately 389.6 µm; however, the nature of the damage was identical. Samples with deposited Ni-P/Si_3_N_4_ nanocomposite layers with different dispersion phase contents were also tested under conditions using MoS_2_ grease. They showed much better tribological properties than aluminum alloy without a deposited coating, because during microscopic observations no cracks, grooves, or damage on the tested surfaces were noticed. The only signs of wear were the sanded tops of individual, point-shaped bumps. Very delicate and hardly noticeable signs of wear of the Ni-P/Si_3_N_4_ surface are shown in Figure 7c,d.

On the basis of the obtained preliminary results and analyses, it was found that the ball-on-disc test can be continued without changing the selected counterspecimen, and the adopted values of load and rotational speed. The modifications were limited to only the time of tests, which was extended. The results of the second stage are presented below.

**Figure 8:**
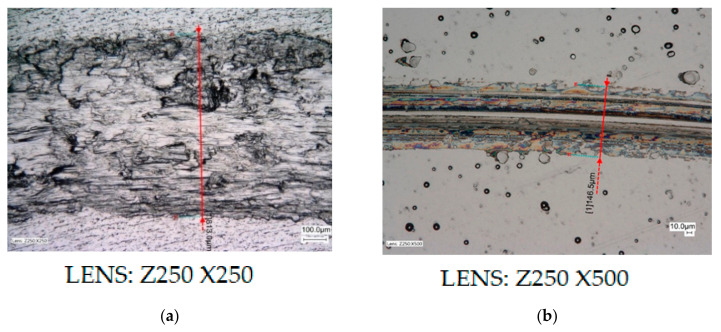

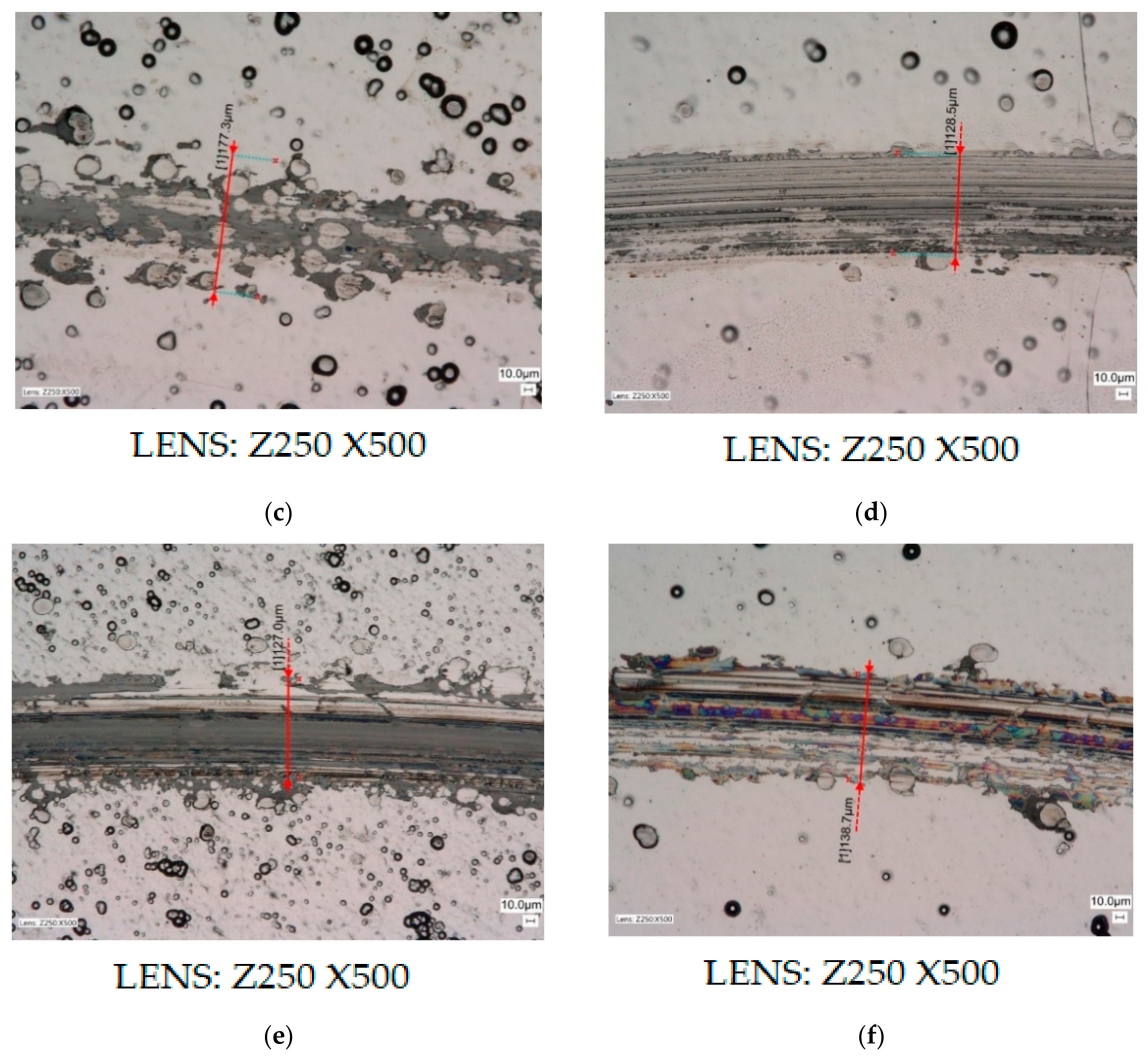
Samples of AW-7075 alloy without coating and with Ni-P and Ni-P/Si_3_N_4_ layers (with MoS_2_ grease). ((**a**)—AW-7075, (**b**)—Ni-P (10 µm), (**c**)—Ni-P (20 µm), (**d**)—Ni-P (30 µm), (**e**)—Ni-P/Si_3_N_4_ (2g), (**f**)—Ni-P/Si_3_N_4_ (5g); crack widths (µm): (**a**)—813.0, (**b**)—146.5, (**c**)—177.3, (**d**)—128.5, (**e**)—127.0, (**f**)—138.7).

The results from the second stage of tribological tests, which were carried out during 300 s (5 min) and with the use of MoS_2_ dry grease, are presented in Figure 8 and in Table 8. All other parameters and wear diameters remained unchanged. Specialized computer software, which is an integral part of the light microscope, was used again. The drawings show high resolution microscopic images showing the widths of the cracks or carved grooves with an accuracy of 0.1 µm.

Based on the results of the second stage tribological tests, which were carried out on all samples, it was noted that the Ni-P and Ni-P/Si_3_N_4_ coatings contribute to significant reductions in surface wear, and are also resistant to damage and grooves compared to the alloy AW-7075 without any deposited coating. In the case of the AW-7075 alloy under examination, the same signs of wear and damage appeared after the preliminary tests, but with the difference that this time the groove widths were clearly larger. The time of the study had an impact on their size.

On the remaining samples, which were covered with Ni-P and nanocomposite layers, signs of wear were noticed only in the form of cracks. The smallest crack width and least wear were observed for the Ni-P/Si_3_N_4_ layer (2 g/dm^3^), for which values were in the range of 127.0 ÷ 134.1 µm, and Ni-P with a thickness of 30 µm, for which the values 128.5 ÷ 133.1 µm were recorded. The content of the dispersion phase contributed to the increase of the layer’s resistance to abrasive wear. However, increasing its content from 2 to 5 g/dm^3^ in the galvanic bath caused a slight increase in surface wear, because the crack width slightly increased to the range of 138.7 ÷ 152.5 µm. The highest wear of the coating surface was noted for samples with a Ni-P layer of the thickness 10, on which the crack width was 146.5–151.1 µm, and 20 µm.

However, in the case of a Ni-P (20 µm) sample, the crack width cannot be clearly determined. The boundary between the part of the area subject to transitions and counterspecimen pressure, and the part that has not been subjected to it, is not clear (blurred). However, after thorough analysis of microscopic images, the crack widths were determined in the range of 177.3–198.5 µm. For all other samples with deposited coatings, the edges of the cracks are very clear and their widths can be explicitly determined.

**Figure 9:**
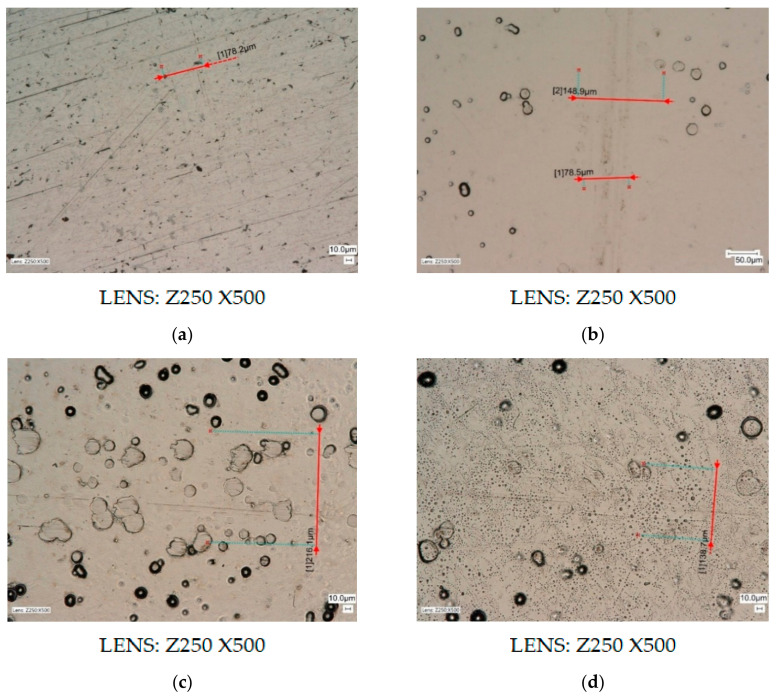

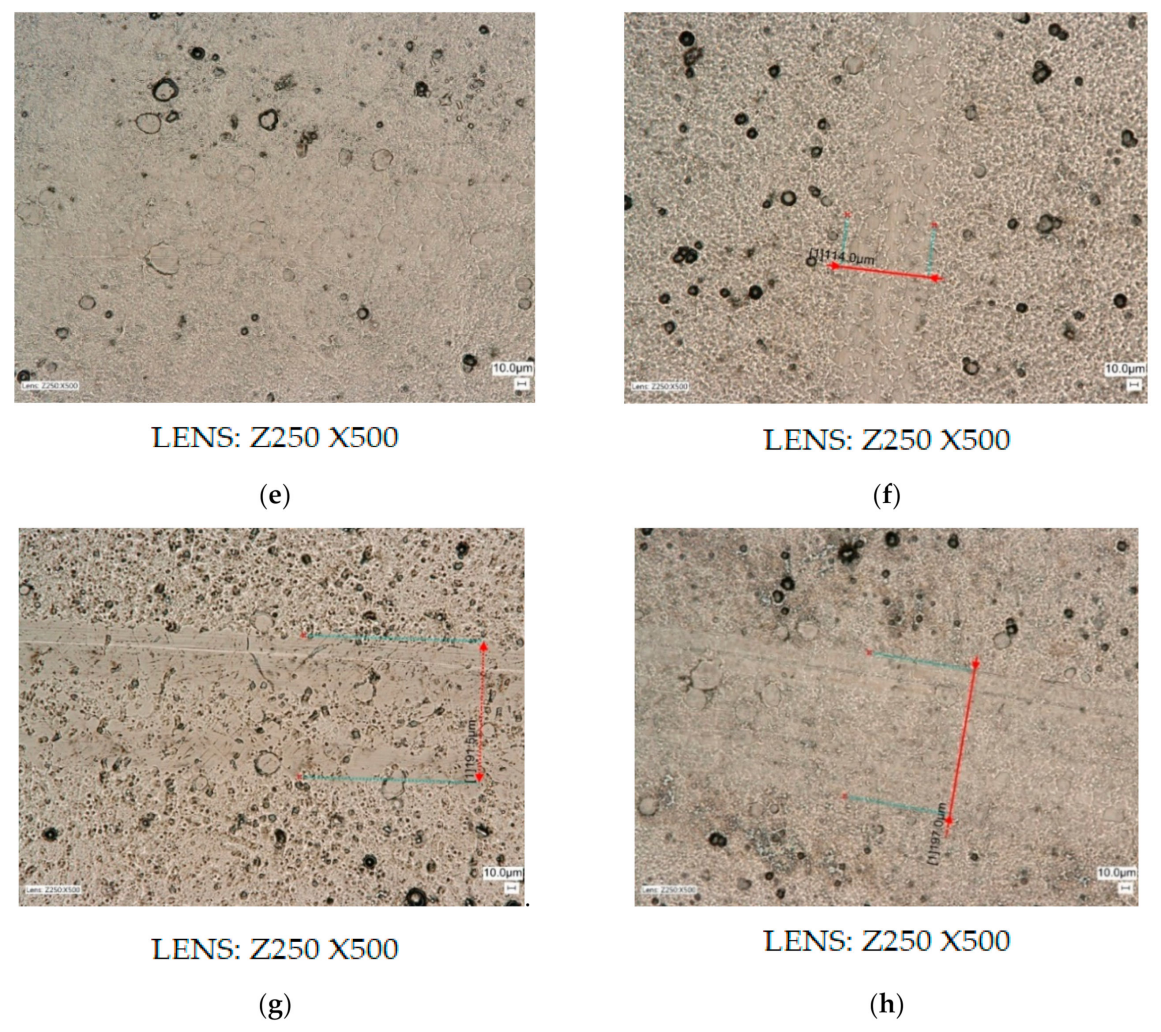
Samples of AW-7075 alloy without coating and with Ni-P and Ni-P/Si_3_N_4_ layers. ((**a**)—AW-7075, (**b**)—Ni-P (10), (**c**)—Ni-P (20), (**d**)—Ni-P (30), (**e**,**g**)—Ni-P/Si_3_N_4_ (2g), (**f**,**h**)—Ni-P/Si_3_N_4_ (5g); crack widths (µm): (**a**)—78.2, (**b**)—148.9, (**c**)—216.1, (**d**)—138.7, (**e**)—no scratches, (**f**)—114.0, (**g**)—191.5, (**h**)—197.0).

**Figure 10:**
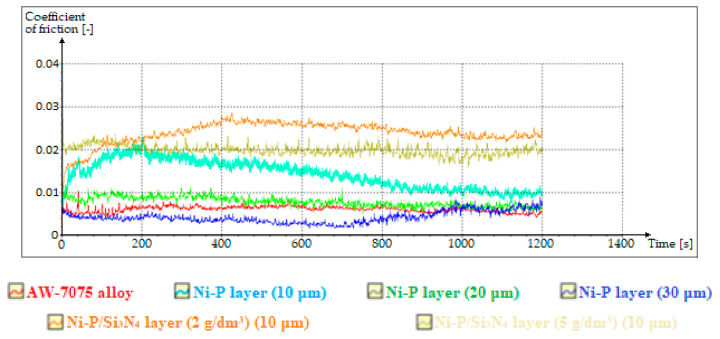
Graph of friction coefficient changes during tribological tests.

Tests of surface resistance to abrasive wear using the ball-on-disc method with the use of semisynthetic gear oil:

The results of the second part of the tribological tests, which were carried out at 5 and 10 N loads, rotational speed 1 s^−1^, during 1200 s (20 min), and with the use of gear oil (GL-4 75W/90), are shown in Figure 9 and Figure 10 and in Table 9. The wear diameter for all samples was 30 mm. The drawings show high resolution microscopic images in which the crack widths are marked with an accuracy of 0.1 µm. Figure 9a–f shows the results of tests carried out at 5 N load.

The microscopic images of the resulting cracks, which are shown in Figure 9, clearly differ from each other. Crack widths characterizing the degree of surface wear depended primarily on the type and conditions of the surface tested, the lubricant used, the counterspecimen pressure force, and the duration of the tests at a set speed of rotation. Crack widths are not the only determinant of surface wear and its behavior in given operating conditions.

In this part, during the tribological tests with the use of semisynthetic gear oil, the values of friction coefficients were measured in parallel, the changes of which are shown in Figure 10. However, starting from a comparison of the resulting cracks after testing with gear oil, very small widths were observed for the AW-7075 sample without coating. However, compared to all other tested samples, which were characterized by a much greater roughness, the aluminum alloy disc had the significant advantage of being very carefully polished to the so-called smooth mirror level, which was confirmed in surface morphology and topography tests. The crack width was in a constant narrow range of 75.8–78.2 µm. Apart from the crack, no surface damage was seen compared to the study in the first part. For samples with deposited Ni-P coatings, the crack widths were different and decreased as the thickness of the layers increased. The crack for 10 μm thick Ni-P layer is clearly visible, and both the crack and the noticeable wear area near the crack are marked. However, in the case of Ni-P layers with thicknesses of 20 and 30 µm, the situation looks completely different, because the wear again consisted mainly in sanding the tops of uneven surfaces, and the cracks are practically invisible. Compared to Ni-P coatings with a thickness of 20 µm, for which the width of the wear trace was from 216.1 to 259.6 µm, for the layers with a thickness of 30 µm this width decreased to values in the range of 137.6–138.7 µm. The sample with a deposited Ni-P/Si_3_N_4_ nanocomposite layer (2 g/dm^3^) showed the highest resistance to crack occurrence and other signs of wear, because during microscopic observations it is difficult to see any traces of a counterspecimen passage. In Figure 9e no signs of wear were noticed.

On the other side of the sample, the crack was also very difficult to notice; however, very carefully, a barely visible trace was marked, whose width could not be accurately determined. With a rough approximation it was set at about 99.5 µm. The sample with the deposited Ni-P/Si_3_N_4_ layer (5 g/dm^3^) showed greater wear compared to the previous sample, because the crack was clear and its width was in the range of 114.0–118.5 µm. It was noticed that despite very small thicknesses of composite coatings (10 µm), these layers were characterized by much higher wear resistance than all Ni-P coatings of different thicknesses (10, 20, and 30 µm), and in the case of Ni-P/Si_3_N_4_ layer (2 g/dm^3^) also against the smoothly polished alloy AW-7075. In order to characterize the nanocomposite layers more accurately, additional tests were carried out with the increased counterspecimen pressure, i.e., 10 N—the results are shown in Figure 9g,h.

Increasing the counterspecimen pressure from 5 to 10 N resulted in the appearance of wider, clearer cracks, whose edges and traces of wear at the places of counterspecimen passage are more visible. Similarly, no damage to the coating surface was noted in the form of cracks, delamination, and material losses or nicks. In the case of samples with Ni-P/Si_3_N_4_ (2 g/dm^3^) and Ni-P/Si_3_N_4_ (5 g/dm^3^) nanocomposite coatings, the crack widths for a given load are similar. However, compared to the previous test, their width increased accordingly: from approximately 99.5 to 191.5–201.4 µm for Ni-P/Si_3_N_4_ (2 g/dm^3^) and from 114.0–118.5 to 197.0–197.4 µm for Ni-P/Si_3_N_4_ (5 g/dm^3^). Increasing the load contributed to increased wear, which was even, but the wear trace was smaller compared to a thicker Ni-P coating (20 µm) without a dispersion phase. In addition, the impact of the dispersion phase content on tribological properties is more noticeable at lower loads. The tests confirmed that nanocomposite layers deposited on the AW-7075 alloy can be subjected to loads of different values, and can also be successfully exploited in various environments. 

All tests confirmed that the tested layers contributed to an increase in the surface resistance to tribological wear to varying degrees.

For a more accurate illustration of the degree of wear, Table 9 summarizes the crack widths for individual samples.

The graph of friction coefficient changes was obtained automatically from tribological tests using computer software attached to the BRUKER UMT TriboLab device. For all samples, the coefficient of friction values stabilized between 1000 and 1200 s of the test. The obtained graph shows that the coefficients of friction for Ni-P layers with thicknesses of 10, 20, and 30 μm, and for aluminum alloy polished to a “smooth mirror,” after working for 1000 s, set at a very similar level in the range of 0.005–0.010. In contrast, nanocomposite Ni-P/Si_3_N_4_ layers with different dispersion phase contents are characterized by the largest friction coefficient, which after 1000 s oscillated in the range of 0.020–0.025, which is reflected in the results of profilometric tests, which confirmed their greatest roughness. However, despite the highest values of friction coefficients observed, the surfaces of Ni-P/Si_3_N_4_ nanocomposite layers showed at the same time the highest resistance to abrasive wear.

## 4. Discussion

The tested nanocomposite coatings had the optimum compositions (chemical and mechanical) for tests of that kind—especially according to the adhesion results of Ni-P and Ni-P/Si_3_N_4_ layers in earlier measuring and research in the field of the mechanical tests of electroless coatings [40]. The results of the presented research constitute a good basis for modifying the properties of details made of aluminum alloys by applying composite alloy layers on their surfaces using the chemical reduction method, which will allow one to increase the functionality of the finished products.

The tested Ni-P and Ni-P/Si_3_N_4_ coatings are characterized by better mechanical and tribological properties than the AW-7075 alloy. Ni-P layers have a higher hardness than the AW-7075 alloy on which they were deposited, and the addition of Si_3_N_4_ nanoparticles caused that the obtained Ni-P/Si_3_N_4_ nanocomposite coatings to demonstrate the highest hardness among all tested layers. The hardness slightly decreases when the content of the dispersion phase increases from 2 to 5 g/dm^3^ in the bath. Ni-P/Si_3_N_4_ nanocomposite coatings are produced in a bath with a dispersion phase content of 2 g/dm^3^ or 5 g/dm^3^, and have better tribological properties compared to Ni-P coatings without a dispersion phase with a thickness of 10 µm. In addition, no erasure or damage to the surfaces of the coatings in the form of cracks, delamination, and losses or nicks of the material was observed during tribological tests. The microscopic images of the resulting cracks clearly differ from each other. Crack widths characterizing the degree of surface wear depended primarily on the type and condition of the surface tested, the lubricant used, the counterspecimen pressure force, and the duration of the tests at a set speed of rotation. Crack widths are not the only determinant of surface wear; however, they are the most important element in ball-on-disc method research. The sample with the deposited Ni-P/Si_3_N_4_ nanocomposite layer (2 g/dm^3^) showed the highest resistance to cracking and other signs of wear. It was also noticed that despite very small thicknesses of Ni-P and composite Ni-P/Si_3_N_4_ coatings, these layers were characterized by much higher wear resistance than the smoothly polished AW-7075 alloy. For samples with Ni-P/Si_3_N_4_ nanocomposite coatings (2 g/dm^3^) and Ni-P/Si_3_N_4_ (5 g/dm^3^), the influences of the dispersion phase content on tribological properties were more noticeable. The content of Si_3_N_4_ nanoparticles has a positive effect on the mechanical and tribological properties of the layers; however, a larger increase in the content of the dispersion phase (from 2 to 5 g/dm^3^) affects it negatively, and both hardness and wear resistance begin to decrease. It is noteworthy that Ni-P/Si_3_N_4_ nanocomposite coatings with a thickness of 10 µm, manufactured in a bath with a phase content of 2 g/dm^3^, have the best abrasion resistance compared to all other tested coatings, despite the highest values of roughness parameters observed and rough peaks in profilometric tests. The tests confirmed that nanocomposite layers deposited on the AW-7075 alloy can be subjected to various loads, and can also be used in various environments, using both gear oil and dry MoS_2_ grease. All tests also confirmed that the tested layers contributed to an increase in the surface resistance to abrasive wear to a varying degree and are good materials for conducting further tribological studies.

## Figures and Tables

**Table 1:** Chemical composition of AW-7075 alloy, percentage by mass.

Chemical Composition [%]										
Zn	Mg	Cu	Fe	Si	Mn	Cr	Zr	Ti	other	Al
5.1–6.1	2.1–2.9	1.2–2.0	≤0.50	≤0.4	≤0.3	0.18–0.28	≤0.25	≤0.20	≤0.05	Rest

**Table 2:** Parameters of tribological tests (the first step).

Stage	Load, N	Time, s	Wear Diameter, mm	Rotation Speed, s^−1^	Counterspecimen	Contact Pressure, MPa
The first-preliminary	7.5	60	15	2	100Cr6 bearing steel ball with a radius of 3.15 mm (1/4”)	0.062 (AW7075) 0.071 (Ni-P) 0.071 (Ni-P/Si_3_N_4_)
The second	7.5	300	15	2	100Cr6 bearing steel ball with a radius of 3.15 mm (1/4”)	0.062 (AW7075) 0.071 (Ni-P) 0.071 (Ni-P/Si_3_N_4_)

**Table 3:** Parameters of tribological tests (the second step).

Stage	Load, N	Time, s	Wear Diameter, mm	Rotation Speed, s^−1^	Counterspecimen	Contact Pressure, MPa
The third	5	1200	30	1	100Cr6 bearing steel ball with a radius of 3.15 mm (1/4”)	0.054 (AW7075) 0.062 (Ni-P) 0.062 (Ni-P/Si_3_N_4_)
The fourth	10	1200	30	1	100Cr6 bearing steel ball with a radius of 3.15 mm (1/4”)	0.066 (AW7075) 0.078 (Ni-P) 0.078 (Ni-P/Si_3_N_4_)

**Table 4:** Content of elements of Ni-P/Si_3_N_4_ layers.

Si_3_N_4_ Content in Bath, g/dm^3^	Chemical Element, % Vol.		
	P	Ni	Si
0	23.09	76.91	-
2	24.08	75.48	0.44
5	24.64	74.78	0.58

**Table 5:** Roughness parameters of AW-7075, Ni-P, and Ni-P/Si_3_N_4_ layers.

Material	Thickness of Layer, µm	Si Content in Layer, % vol.	Si_3_N_4_ Content in Bath, g/dm^3^	Rz, μm	Standard Deviation	Ra, μm	Standard Deviation
AW-7075	-	-	-	0.358	0.0513	0.0456	0.0029
Ni-P	10	-	-	1.95	0.638	0.113	0.0257
	20	-	-	2.61	0.693	0.190	0.0523
	30	-	-	1.32	0.589	0.101	0.0313
Ni-P/Si_3_N_4_	10	0.44–0.48	2	14.1	3.45	1.54	0.481
	10	0.58	5	2.72	0.926	0.146	0.0512

**Table 6:** Directions of surface structures.

Material	Thickness of Layer, µm	Si_3_N_4_ Content in Bath, g/dm^3^	Isotropy, %	The First Direction, [⁰]	The Second Direction, [⁰]	The Third Direction, [⁰]
AW-7075	-	-	26.3	0.238	90.0	69.3
Ni-P	10	-	66.8	169	163	89.4
	20	-	66.3	90.0	135	45.0
	30	-	17.2	45.0	90.0	135
Ni-P/Si_3_N_4_	10	2	81.5	45.0	90.0	135
	10	5	66.9	135	90.0	26.5

**Table 7:** Microhardness of layers.

Material	Thickness of Layer, µm	Si Content in Layer, % vol.	Si_3_N_4_ Content in Bath, g/dm^3^	HV0,03	Standard Deviation
AW-7075	-	-	-	192.46	6.69
Ni-P	10	-	-	590.62	19.44
	20	-	-	627.83	21.13
	30	-	-	622.34	4.58
Ni-P/Si_3_N_4_	10	0.44–0.48	2	639.97	18.05
	10	0.58	5	632.76	17.98

**Table 8:** Lists the crack widths for individual samples (the first step).

Material	AW-7075	Ni-P (10 µm)	Ni-P (20 µm)	Ni-P (30 µm)	Ni-P/Si_3_N_4_ (10 µm, 2 g/dm^3^)	Ni-P/Si_3_N_4_ (10 µm, 5 g/dm^3^)
Crack widths, µm	769.6–813.0	146.5–151.1	177.3–198.5	128.5–133.1	127.0–134.1	138.7–152.5

**Table 9:** Crack widths after tribological tests (the second step).

	AW-7075	Ni-P (10 µm)	Ni-P (20 µm)	Ni-P (30 µm)	Ni-P/Si_3_N_4_ (10 µm, 2 g/dm^3^)	Ni-P/Si_3_N_4_ (10 µm, 5 g/dm^3^)	Ni-P/Si_3_N_4_ (10 µm, 2 g/dm^3^)	Ni-P/Si_3_N_4_ (10 µm, 5 g/dm^3^)
Force, N	5	5	5	5	5	5	10	10
Crack widths, µm	75.8–78.2	148.9–172.2	216.1–259.6	137.6–138.7	0–99.5	114.0–118.5	191.5–201.4	197.0–197.4

## References

[1] Burakowski T. (2004). Rozważania o Synergizmie w Inżynierii Powierzchni.

[2] Burakowski T. (2013). Areologia Podstawy Teoretyczne.

[3] Kupczyk M. (2009). Wytwarzanie i Eksploatacja Narzędzi Skrawających z Powłokami Przeciwzużyciowymi.

[4] Trzaska M. (2010). Studies of the structure and properties of Ni-P and Ni-P/Si3N4 surface layers deposited on aluminum by the electroless method. J. Achiev. Mater. Manuf. Eng..

[5] Blicharski M. (2012). Inżynieria Powierzchni.

[6] Starosta R., Dyl T. (2008). Obróbka Powierzchniowa.

[7] Chronowska-Przywara K., Kot M., Zimowski S. (2014). The research techniques for analysis of mechanical and tribological properties of coating-substrate systems. Zesz. Nauk. Politech. Śląskiej.

[8] Śmierzchalski D., Wieczorowski M. (2013). Simple scratch method for industry and for teaching. Inżynieria Masz..

[9] Chronowska-Przywara K., Kot M. (2014). Effect of scratch test parameters on the deformation and fracture of coating-substrate systems. Tribologia.

[10] Shilong W., Xuefei H., Mengixiao G., Weigang H. (2015). Microstructure and mechanical properties of Ni-P-Si3N4 nanowire electroless composite coatings. Appl. Surf. Sci..

[11] Farzeneh A., Mohammadi M., Ehteshamzadeh M., Mohammadi F. (2013). Electrochemical and structural properties of electroless Ni-P-SiC nanocomposite coatings. Appl. Surf. Sci..

[12] Karthikeyan S., Ramamoorthy B. (2014). Effect of reducing agent and nano Al_2_O_3_ particles on the properties of electroless Ni-P coating. Appl. Surf. Sci..

[13] Balaraju J.N., Ezhil S.V., Rajam K.S. (2010). Electrochemical behavior of low phosphorus electroless Ni-P-Si_3_N_4_ composite coatings. Mater. Chem. Phys..

[14] Franco M., Sha W., Aldic G., Malinov S., Cimenoglu H. (2016). Effect of reinforcement and heat treatment on elevated temperaturę sliding of electroless Ni-P/SiC composite coatings. Tribol. Int..

[15] Soleimani R., Mahboubi F., Arman S.Y., Kazemi M., Maniee A. (2015). Development of mathematical model to evaluate microstructure and corrosion behavior of electroless Ni–P/nano-SiC coating deposited on 6061 aluminum alloy. J. Ind. Eng. Chem..

[16] Matik U. (2016). Structural and wear properties of heat-treated electroless Ni-P alloy and Ni-P-Si3N4 composite coatings on iron based compacts. Surf. Coat. Technol..

[17] Sudagar J., Venkateswarlu K., Lian J. (2010). Dry sliding wear properties of a 7075-T6 aluminum alloy coated with Ni-P (h) in different pretreatment conditions. J. Mater. Eng. Perform..

[18] Vijayanand M., Elansezhian R. (2014). Effect of different pretreatments and heat treatment on wear properties of electroless Ni-B coatings on 7075-T6 aluminum alloy. Procedia Eng..

[19] Prasanta S., Suman K.D. (2011). Tribology of electroless nickel coatings—A review. Mater. Des..

[20] Sudagar J., Lian J., Sha W. (2013). Electroless nickel, alloy, composite and nano coatings—A critical review. J. Alloys Compd..

[21] Balaraju J.N., Seshadri S.K. (1999). Preparation and characterization of electroless Ni-P and Ni-P-Si_3_N_4_ composite coatings. Trans. Inst. Met. Finish..

[22] Mazurek A., Cieślak G., Bartoszek W., Trzaska M. (2017). Abrasion resistance of Ni-B/Si_3_N_4_ composite layers produced by electroless method. Arch. Mater. Sci. Eng..

[23] Trzaska M., Cieślak G., Mazurek A. (2016). Structure and properties of Ni-P/PTFE composite coatings produced by chemical reduction method. Compos. Theory Pr..

[24] Li Z., Farhat Z. (2020). Hertzian Indentation Behavior of Electroless Ni-P-Ti Composite Coatings. Metall. Mater. Trans. A.

[25] Varshney P., Chhangani S., Prasad M., Pati S., Gollapudi S. (2020). Effect of grain boundary relaxation on the corrosion behaviour of nanocrystalline Ni-P alloy. J. Alloys Compd..

[26] Sharifalhoseinia Z., Entezaria M.H., Davoodib A., Shahidi M. (2020). Access to nanocrystalline, uniform, and fine-grained Ni-P coating with improved anticorrosive action through the growth of ZnO nanostructures before the plating process. Corros. Sci..

[27] Kanamori K., Kimoto Y., Toriumi S., Yonezu A. (2020). Evaluation of adhesion durability of Ni–P coating using repeated Laser Shock Adhesion Test. Surf. Coat. Technol..

[28] Touri S., Monirvaghef S.M. (2020). Fabrication and characterization of functionally graded Ni-P electroless coating with variable properties along the surface of the coating. Mater. Today Commun..

[29] Mohsenifar F., Ebrahimifar H. (2020). Effect of titanium oxide ceramic particles concentration on microstructure and corrosion behaviour of Ni–P–Al_2_O_3_–TiO_2_ composite coating. Bull. Mater. Sci..

[30] Liu Y., Zhang L., Zhao W., Sheng H., Li H. (2020). Fabrication and properties of carbon fiber-Si3N4 nanowires-hydroxyapatite/phenolic resin composites for biological applications. Ceram. Int..

[31] Khullar P., Zhu D., Gilbert J.L. (2020). Fretting corrosion of Si_3_N_4_ vs. CoCrMo femoral heads on Ti-6Al-V trunnions. J. Orthop. Res..

[32] Cao S., Zhang D., Wang J., Zhang J., Zhou J., Zhang Y. (2020). Synthesis of self-toughness porous Si_3_N_4_ ceramics with three-dimensional cage structures. Mater. Lett..

[33] Zhang J., Liu J., Wang Z., Chen W., Hu B., Zhang Y., Liao H., Ma S. (2020). Tribological behavior and lubricating mechanism of Si_3_N_4_ in artificial seawater. Ceram. Int..

[34] Nassajpour-Esfahani A., Emadi R., Alhaji A., Bahrami A., Haftbaradaran-Esfahani M. (2020). Towards high strength MgAl_2_O_4_/Si_3_N_4_ transparent nanocomposite, using spark plasma sintering. J. Alloys Compd..

[35] Yan L., Liu J., Wang X., Ma C., Zhang C., Wang H., Wei Y., Wen X., Yang Y., Li Y. (2020). Ru catalysts supported by Si_3_N^4^ for Fischer-Tropsch synthesis. Appl. Surf. Sci..

[36] Wang L., Qi Q., Yang X., Zhang H., Liu Z., Ge S., Peng X., Liu L., Liu Y., Liu X. (2020). Mechanical properties optimization of Si_3_N_4_ ceramics by in-situ introduction of core-shell structural W-Fe_5_Si_3_. Compos. Part B.

[37] Szala M., Kot E.A. (2017). Influence of repainting on the mechanical properties, surface topography and microstructure of polyester powder coatings. Adv. Sci. Technol. Res. J..

[38] Trzaska M. (2014). Structure and properties of composite Ni-P/SiC surface layers produced by chemical reduction on aluminum and its alloys. Przem. Chem..

[39] Czapczyk K., Legutko S., Siwak P., Grochalski K., Mazurek A. (2018). The influence of thickness of the Ni-P layers deposited on the AW-7075 aluminum alloy on their adhesion and mechanical properties. Inżynieria Powierzchni Surf. Eng..

[40] Czapczyk K., Legutko S., Siwak P., Gapiński B., Cieślak G. (2018). Mechanical properties of Ni-P/Si_3_N_4_ nanocomposite surface leyers produced by chemical reduction on AW-7075 aluminum alloy. Przem. Chem..

